# A randomized, sham-controlled clinical trial to evaluate the NET Device™ for reducing withdrawal symptom severity during opioid discontinuation

**DOI:** 10.3389/fpsyt.2025.1510428

**Published:** 2025-02-19

**Authors:** Mark K. Greenwald, Cynthia L. Arfken, Joe R. Winston

**Affiliations:** ^1^ Department of Psychiatry and Behavioral Neurosciences, Wayne State University School of Medicine, Detroit, MI, United States; ^2^ NET Recovery Corp™ (NRC), Wilmington, DE, United States

**Keywords:** opioid use disorder, detoxification, withdrawal, craving, transcranial electrical stimulation, tACS

## Abstract

**Background:**

Neuromodulation is a promising approach for opioid discontinuation, as not all patients with opioid use disorder (OUD) seek pharmacotherapy. The NET Device is a non-invasive, battery-powered, portable, device that provides bilateral, transcranial, transcutaneous, alternating current stimulation (tACS) for patients experiencing opioid withdrawal. This clinical trial prospectively evaluated whether NET Device utilization is effective for persons with OUD undergoing opioid discontinuation without medications for OUD (MOUD).

**Methods:**

This randomized, sham-controlled trial was conducted at a single residential treatment center. Persons with OUD undergoing opioid discontinuation were assigned to active or sham device treatment. Clinical Opiate Withdrawal Scale (COWS) total scores were measured prior to and during device use. We tested whether active stimulation would produce a clinically meaningful (≥15%) decrease in COWS score from baseline to 1-hr post-stimulation, compared to sham.

**Results:**

108 participants (55 sham, 53 active; 59.3% male, 89.8% white; 71.3% fentanyl-positive) form the intent-to-treat dataset. Mean ( ± 1 SD) COWS score in the active device group decreased from baseline (18.1 + 4.4) to 1-hr (7.0 + 4.1); this 61.3% decrease (*d*=2.14) exceeded the pre-specified 15% criterion. COWS scores decreased more for active (-11.1 ± 5.2) than sham (-8.8 ± 6.3), *p*<.05, *d*=-.41. A higher proportion of participants in active *vs*. sham showed ≥15% reduction in COWS (98.1% *vs*. 83.6%), *p*=.016. Device utilization was longer for active than sham, 43.9 + 46.2 *vs*. 30.0 + 39.2 hours, *p*=.008, and fewer participants requested MOUD (26% *vs*. 49%, p<0.02).

**Conclusion:**

The NET Device is effective, safe and well-tolerated for reducing opioid withdrawal symptoms. This device received FDA market clearance in May 2024.

**Clinical trial registration:**

ClinicalTrials.gov, identifier NCT04916600.

## Introduction

Nonmedical and illicit opioid use, and opioid use disorder (OUD) continue to threaten societal, health and economic welfare across North America (1–6), fueled by exposure to synthetic opioids and psychostimulants (7–13). These factors, including the protracted rise in opioid-related overdose deaths (14) and emergency department visits (15, 16), have motivated assertive and widespread initiatives to combat this crisis.

Unfortunately, OUD treatment participation in the US remains disturbingly low. In 2021, about 2.5 million adults had past-year OUD, but only about 35.6% of these individuals received any past-year treatment (17) due to several barriers (18–21), and utilization of medications for OUD (MOUD) was estimated at 22.2% (17). Notably, there are major sociodemographic disparities in who receives MOUD: those more likely to receive MOUD tend to have more severe past-year OUD, receive treatment via telehealth, live in metropolitan areas, report family income < $50,000, and are male and non-Hispanic White (17). Furthermore, a sizable proportion of patients entering OUD treatment express reservations about MOUD (e.g. side effects, inconvenience, stigma) and express curiosity about non-medication interventions for transitioning to longer-term abstinence (22–27), although presently such options are sparse. This lack of confidence in treatment involving medications could lead many OUD patients to avoid or drop out of otherwise lifesaving treatment.

Medical devices offer an emerging alternative therapeutic approach that could be useful in managing the transition from illicit opioid use to abstinence (28). NeuroElectric Therapy™ (NET^®^) is a promising neurostimulation modality for attenuating withdrawal symptoms in persons with OUD undergoing opioid discontinuation but, until now, has not undergone rigorous efficacy evaluation. Earlier-generation versions of this device have been studied extensively under open-label, non-controlled conditions, in the inpatient setting, as a possible monotherapy for medication-free discontinuation from chronic substance use (29–32). Results from observational pilot studies in Europe and the US indicate that treatment with the NET Device as monotherapy, i.e. without MOUD or adjunctive medications, can rapidly decrease opioid discontinuation-related drug craving and withdrawal symptom elevations. Yet, those pilot studies did not use intent-to-treat (ITT), randomization, sham-control, or blinded procedures. The present study is a prospective, randomized, sham-controlled, blinded trial that is intended to address these limitations and meet the FDA conditions for market clearance.

The primary study objective was to determine whether use of the NET Device reduces opioid withdrawal symptom severity in persons with OUD experiencing moderate or greater symptoms of opioid withdrawal at baseline. We hypothesized that active device use would provide a clinically meaningful decrease in opioid withdrawal symptom severity for most patients, which would be significantly greater than for the sham group.

## Materials and methods

The Wayne State University Institutional Review Board approved this trial August 23, 2021. The first participant enrolled on November 24, 2021, and the final participant concluded on July 07, 2023.

### Study design

This randomized, single-site trial at four facilities used a superiority design to compare outcomes following active device vs. sham control treatment. **Supplementary Figure S1** illustrates the overall study schema and **Supplementary Figure S2** illustrates the schedule of activities (which includes post-discharge outpatient 12-week follow-up data, to be reported separately).

The minimum 1-hr device treatment period was selected to ensure a controlled degree of exposure to the device (active or sham) for all participants and based on the predicate device; in earlier open-label studies, the active NET Device produced benefits in about 15-20 min. Completing this 1-hr period triggered follow-up protocol assessments. Furthermore, efficacy of the predicate device was also measured at 1-hr after starting stimulation. The maximum 7-day device treatment period was chosen to limit variability in the duration of exposure.

### Setting

All participants were screened, enrolled, and underwent evaluation at four residential addiction treatment facilities within the same organization: two for males only and two for females only. Typically, clients are admitted for 28 days, although some leave earlier. Senior management, therapists, nursing staff, and core services are common across all locations. The admissions process, medical and clinical assessments occurred separately for males and females at their respective facilities.

### Participant screening and selection

For ethical reasons, recruitment and screening occurred as quickly as possible after admission, prior to emergence of moderate-severity opioid withdrawal. Screening included obtaining informed consent, HIPAA authorization, assessment of demographics, contact data, physical exam, pregnancy testing, contraception methods, medical and drug history, and urine drug testing (including amphetamines, barbiturates, buprenorphine, benzodiazepines, cocaine, fentanyl, MDMA, methamphetamine, morphine, methadone, opioids, oxycodone, phencyclidine, and Δ^9^-tetrahydrocannabinol). Assessments were aligned *a priori* with the treatment facility’s standard of care such that electronic case report forms (eCRFs) could be rapidly reviewed for study eligibility by the remote investigator (author MKG), given the time-sensitive nature of enrollment.

During the recruitment and informed consent process, participants were repeatedly told they could receive FDA-approved MOUD at the treatment facility instead of participating in the study. They were also told that upon discontinuation of device stimulation, or if they dropped out of the study, they could receive TAU (including MOUD) at any time. All participants were issued verbal and written “loss-of-opioid tolerance” warnings (i.e. stopping opioids leads to reduced tolerance, and subsequent opioid use increases their risk of overdose and death) which they had to sign. Participants were also provided a naloxone kit upon discharge from the treatment facility and educated on its use. All of these procedures were documented in the medical record and CRFs.

**Supplementary Table S1** lists study inclusion and exclusion criteria. Patients admitted for OUD, between the ages 18-65 years old, in good general health, seeking opioid discontinuation at the treatment facility, self-reporting that they wished to become abstinent without using MOUD, were eligible to participate. Additionally, they had to agree to provide informed consent, follow study procedures, and use medically-accepted highly effective contraception. Prior to receiving either active or sham treatment, all participants who enrolled had to exhibit at least moderate withdrawal severity, operationally defined as a total score of 13 or greater on the Clinical Opiate Withdrawal Scale (COWS) (33). Exclusion criteria included pregnancy or lactation, serious current psychiatric disorder (schizophrenia, bipolar) or use of neuropsychiatric medications that may overlap with NET’s proposed mechanisms of action (e.g. anxiolytics, antidepressants, anticonvulsants, sedating H_1_-receptor antihistamines, prescription or over-the-counter stimulants), need for detoxification from alcohol or benzodiazepines, past 300-day exposure to extended-release buprenorphine, certain chronic illnesses (especially seizures), unstable medical conditions, or presence of cardiac pacemaker.

### Intervention

The NET Device delivers alternating current via surface electrodes placed transcranially (bilaterally) on the mastoid processes (**Supplementary Figure S3**). The device delivers multiple low-amperage waveforms at controlled frequencies and pulse widths that vary throughout each treatment day, with no net direct current component.

In prior clinical studies, the output waveform was optimized for dynamic variations in skin impedance, electrode conductance, frequency and pulse-width related sensation, and orthogonal electrode pressure (e.g. from head pressure when sleeping), leading to improved rates of patient tolerability. Furthermore, human and animal studies, and clinical observation, have identified different electronic waveforms corresponding to subtypes of polysubstance use. These waveforms were programmed into the device at treatment entry based on each participant’s drug screen results at the time of treatment initiation. Stimulation was continuously available (except when bathing) for up to 7 days via transcutaneous electrodes of size approximately 1cm x 2cm. Stimulation output frequency varied from 4 to 3000 Hz and pulse width from 7 to 750 microseconds. Stimulation output current varied from 0 to 3.2 mA (peak) into a 15 kOhm load, and output voltage varied from 0 to 44 volts (peak to peak). Treatment was self-administered, and participants were instructed that they could control the device output intensity and duration according to perceived benefit.

The research study was classified as a Non-Significant Risk (NSR) medical device study in accordance with FDA guidelines. However, safety of the NET Device has not been established for persons who: are pregnant, breastfeeding, or <18 years old; have serious heart conditions or a cardiac pacemaker; have suffered a stroke, brain tumor, or brain injury; have current epilepsy; are suffering serious psychotic illness; or are taking medications such as neurotransmitter blockers. Therefore, these characteristics were part of the exclusion criteria.

Sham treatment, to control for placebo effects, was designed to minimize treatment assignment recognition by the sponsor, principal investigator, independent study monitor, participants, research assistants, and treatment staff. The active and sham interventions both used the NET Device, but the sham intervention used lead wires that (although visually the same as active wires) were rendered non-conductive beforehand, preventing any electrical stimulation from being delivered to the participant.

Participants in the active and sham arms received identical instructions, equipment, electrode attachment methods and locations, and daily reviews of device operation. The apparatus presented both active- and sham-assigned participants with visual cues from the device’s “heartbeat” indicator (a blinking green light-emitting diode which indicated the device was active) during treatment. Research staff instructed each participant that the equipment was designed to be self-administered, that s/he could set the level of stimulation wherever it is comfortable, that stimulation is not always (and does not need to be) sensate, and that device use could be discontinued when the participant felt it was not providing additional benefit.

All participants received treatment as usual (TAU), except MOUD, which they elected (as part of informed consent) not to receive as a condition of study inclusion. During the residential stay, clinical deterioration could potentially occur due to discontinuation of illicit opioids, prescribed opioid agonist medications (buprenorphine, methadone), or other illicit substances. Participants were told that device use (active or sham treatment) would be self-administered, that they could discontinue device use at any time and for any reason, and could receive TAU for their clinical condition including MOUD and comfort medications.

### Randomization and blinding

The biostatistician created the randomization assignment using a 1:1 (active: sham) allocation ratio and, within each treatment group, with stratification by sex (male/female). Only the biostatistician and a research assistant (backup person) could access the randomization codebook, which was never required.

Before study initiation, the biostatistician allocated a random and unique Study Device Number (SDN) to each identical-looking active and sham lead wire, applying pre-printed SDN heat shrink labels. At the study site, the research assistant was notified electronically of participant inclusion and selected a device and lead wires by SDN from a pre-printed randomization table for delivery of active or sham treatment. At the end of the study, all lead wires were delivered to the biostatistician for verification against the group assignment by measuring conductivity using the sponsor-supplied validation circuit.

### Outcome measures

When the trial design was initially described, reduction in opioid withdrawal severity was the secondary efficacy endpoint and the primary endpoint was long-term abstinence without MOUD (34). After data collection was completed, but before unblinding occurred, discussions with the US Food and Drug Administration (FDA) led the sponsor to pursue the 510(k) clearance pathway for the NET Device, which allows clearance of new devices that demonstrate “substantial equivalence” with devices already marketed legally for the same indication for use. For the intervention in this study, the predicate device was the Sparrow Therapy System, referencing data from a single-arm study showing a 15% decline in opioid withdrawal symptom severity (35). Thus, the design and primary outcome for this study were changed to reducing opioid withdrawal symptom severity in the active group only, with secondary outcomes being the withdrawal symptom comparison by treatment assignment.

#### Primary efficacy measure

The primary efficacy endpoint was the total score on the Clinical Opiate Withdrawal Scale (COWS) (33) which is a valid and reliable measure of opioid withdrawal symptom severity. Research staff received training and supervision on this measure before and periodically throughout the study.

#### Safety measures

Safety was assessed through spontaneous reports and systematic interviews regarding proactively identified adverse events of special interest and of concern for this specific study population (AESIs). Interviews were conducted daily for the first 7 days, and then weekly during inpatient and outpatient phases regarding AESIs and with open unstructured questions to solicit unanticipated effects. Adverse events were logged in case report forms, tabulated by the Study Monitor, and reviewed with the Principal Investigator. Documentation and procedures were consistent throughout the study. Research Assistants received robust formal training including 3 days of classroom training covering the clinical protocol, onsite procedures, offsite procedures, investigational device use and management, and eCRF tool configuration, use, and management. The secondary safety endpoint was the prevalence of all adverse events (AEs), serious adverse events (SAEs), adverse device effects (ADEs), serious adverse device effects (SADEs), unanticipated adverse device effects (UADEs), and device deficiencies.

#### Supportive measures

Device treatment perception was assessed at 1-hr post-stimulation using a 1-5 Likert rating scale (1=very confident it is not real stimulation, 2=somewhat confident it is not real stimulation, 3=not sure, 4=somewhat confident it is real stimulation, 5=very confident it is real stimulation).

Acceptability of device use was assessed using complementary approaches. First, participants were asked about their device satisfaction (ratings at 1-hr post-stimulation) based on a single Likert-type question (1=very dissatisfied, 2=somewhat dissatisfied, 3=neither satisfied nor dissatisfied, 4=somewhat satisfied, 5=very satisfied). Second, using two separate Likert scale ratings (1=very unlikely, 2=somewhat unlikely, 3=not sure, 4=somewhat likely, 5=very likely) participants assessed their willingness to (a) personally use this therapeutic approach, and (b) recommend this approach to other persons undergoing opioid discontinuation. Third, we recorded the objective duration (hr) of device utilization. This behavioral approach indicates the extent to which participants use active or sham NET in the absence of concomitant medication to help manage signs and symptoms of opioid withdrawal.

Treatment retention was measured after 1 hour and at specified follow-up intervals. Self-stimulation intensity (in 5-min bins), duration, and device on/off data from each NET Device were automatically transmitted to a computer server and processed offline. Opioid withdrawal symptoms, craving severity using the 3-item Opioid Craving Scale (36), and affect using the 10-item Positive and Negative Affect Scale (37) were measured daily during the first inpatient week. The Depression Anxiety and Stress Scale (38) was administered at baseline and the conclusion of the inpatient phase (33).

### Statistical analysis

Sample size (*n*= 50 per group) was determined for the original study design assessing efficacy of the intervention on long-term abstinence without MOUD (33). As the study objectives were not changed until after conclusion of data collection, sample size was not recalculated but presumed to be adequate as the predicate device had only 26 participants to show efficacy in reducing opioid withdrawal symptoms.

The final sample size was impacted by the inadvertently omitted word (“sponsor”) from the HIPAA authorization form which was discovered during the conduct of the trial. The IRB required reconsenting 77 individuals. For 28 individuals who were not contacted, all data except information existing at randomization (i.e., date, sex, treatment assignment) were purged. The 28 individuals had similar treatment assignment, sex, and month of consenting as the 49 who were reconsented.

For the new design, we evaluated efficacy and safety endpoints during opioid discontinuation within an inpatient setting. The intent-to-treat sample (ITT) included everyone randomized and the per-protocol sample was the subset who: (1) presented with a COWS score of moderate or above; and (2) received at least 1-hr device treatment.

Baseline characteristics were examined by facility and by treatment assignment using *t*-tests and chi-square tests. Prior to evaluating the primary outcome, we examined whether baseline COWS scores could be pooled, using analysis of variance with facility as a factor. For the secondary outcome the poolability analysis incorporated facility and treatment assignment as factors along with their interaction in the model.

To test the primary outcome, we first calculated the percentage difference in baseline to 1-hr COWS total scores only in the active treatment group and then tested whether it was greater than or equal to 15% using a test of proportion. Then, in the second pre-specified analysis, which was contingent on meeting the first threshold (39), the active *vs*. sham group mean difference in COWS baseline to 1-hr change was compared with chi-square using a two-tailed α= .025 to correct for multiple comparisons.

We then conducted supportive testing using t-tests for continuous COWS and subgroup analysis stratified by sex (male/female) and non-opioid SUD (presence/absence) to assess for consistency of findings by demographic and clinical characteristics.

To assess the effectiveness of blinding in this study, we used the James Blinding Index (40). This index is sensitive to the degree of disagreement (rather than the degree of agreement), by placing the highest weight on ‘do not know’ responses. The index ranges from 0 to 1, with 0 being total lack of blinding, 1 being complete blinding and 0.5 being completely random blinding. If the upper bound of the confidence interval is less than 0.5, the study is regarded as lacking blinding (41).

## Results

### Participant characteristics

**Figure 1:**
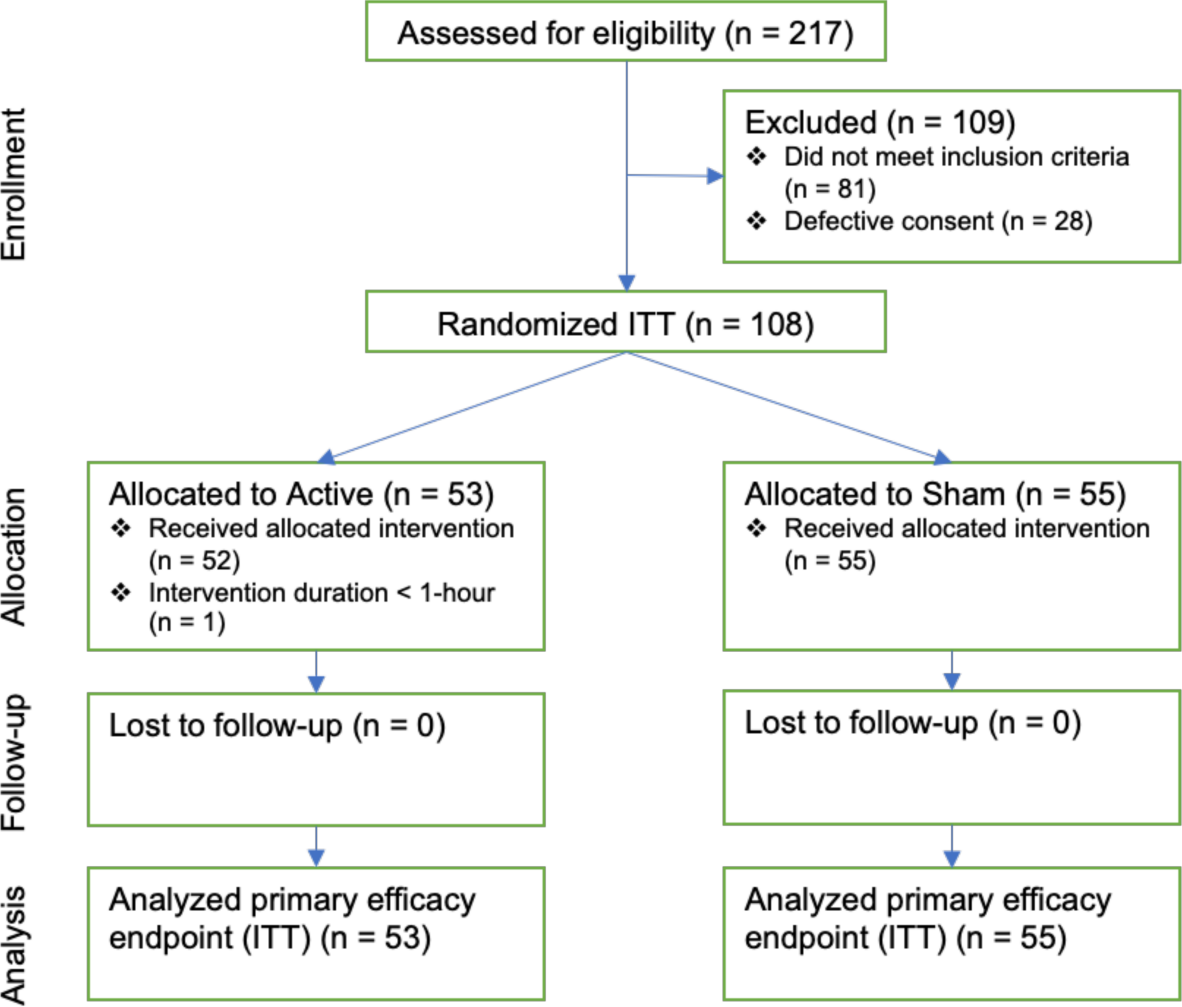
CONSORT diagram.

**Figure 1** presents the CONSORT diagram, which describes participant flow through the study. A total of 136 individuals consented to participate. Of these, 28 signed an earlier version of the consent form (for which the IRB later required revision to alter HIPAA language) but could not be reached for re-consent using the new form; the IRB required that these individuals be excluded. Thus, 108 participants (53 active NET and 55 sham; 59.3% male, 89.8% white; 71.3% fentanyl-positive, 50.0% stimulant [mostly methamphetamine], and 88.9% Δ^9^-THC-positive UDS at screening) form the ITT dataset for testing the efficacy endpoints. One participant in the active group dropped out before 1-hr of device utilization and was excluded from the per-protocol analysis.

**Table 1** presents the demographic and clinical characteristics of the study population. There were no statistical differences between treatment group characteristics. Few participants (6.5%) had only OUD recorded at admission. **Supplementary Table S2** lists sample characteristics, by treatment facility. Apart from sex (which was a stratification factor in the randomization because two facilities housed males, and the other two facilities housed females), the subgroups were similar on demographics by facility.

**Table 1:** Demographic and clinical characteristics by treatment assignment.

	Total sample	Active	Sham
	*N*= 108	*n*= 53	*n*= 55
Sex			
Female	44 (40.7%)	22 (41.5%)	22 (40.0%)
Male	64 (59.3%)	31 (58.5%)	33 (60.0%)
Race			
White	97 (98.8%)	48 (90.6%)	49 (89.1%)
Black	6 (5.6%)	2 (3.8%)	4 (7.3%)
Other	5 (4.6%)	3 (5.7%)	2 (3.6%)
Ethnicity			
Hispanic	3 (2.8%)	1 (1.9%)	2 (3.7%)
Not Hispanic	103 (97.2%)	51 (98.1%)	52 (96.3%)
*Unknown*	2	1	1
Pediatric			
18-20	5 (4.6%)	2 (3.8%)	3 (5.5%)
21+	103 (95.4%)	51 (96.2%)	52 (94.5%)
*Missing*	0	0	0
Age			
Mean, SD	34.1 (8.4)	34.7 (7.6)	33.4 (9.1)
*Missing*	0	0	0
Length of residential stay			
<20 days	51 (47.7%)	22 (42.3%)	29 (52.7%)
20+ days	56 (52.3%)	30 (57.7%)	26 (47.3%)
*Missing*	1		
Primary SUD			
Opioid	88 (81.5%)	42 (79.2%)	46 (83.6%)
Other	20 (18.5%)	11 (20.8%)	9 (16.4%)
Poly-SUD			
Yes	101 (93.5%)	50 (94.3%)	51 (92.7%)
No	7 (6.5%)	3 (5.7%)	4 (7.3%)
COWS total, baseline			
Moderate	98 (90.7%)	48 (90.6%)	50 (90.9%)
Severe	10 (9.3%)	5 (9.4%)	5 (9.1%)

### Efficacy analysis

**Figure 2:**
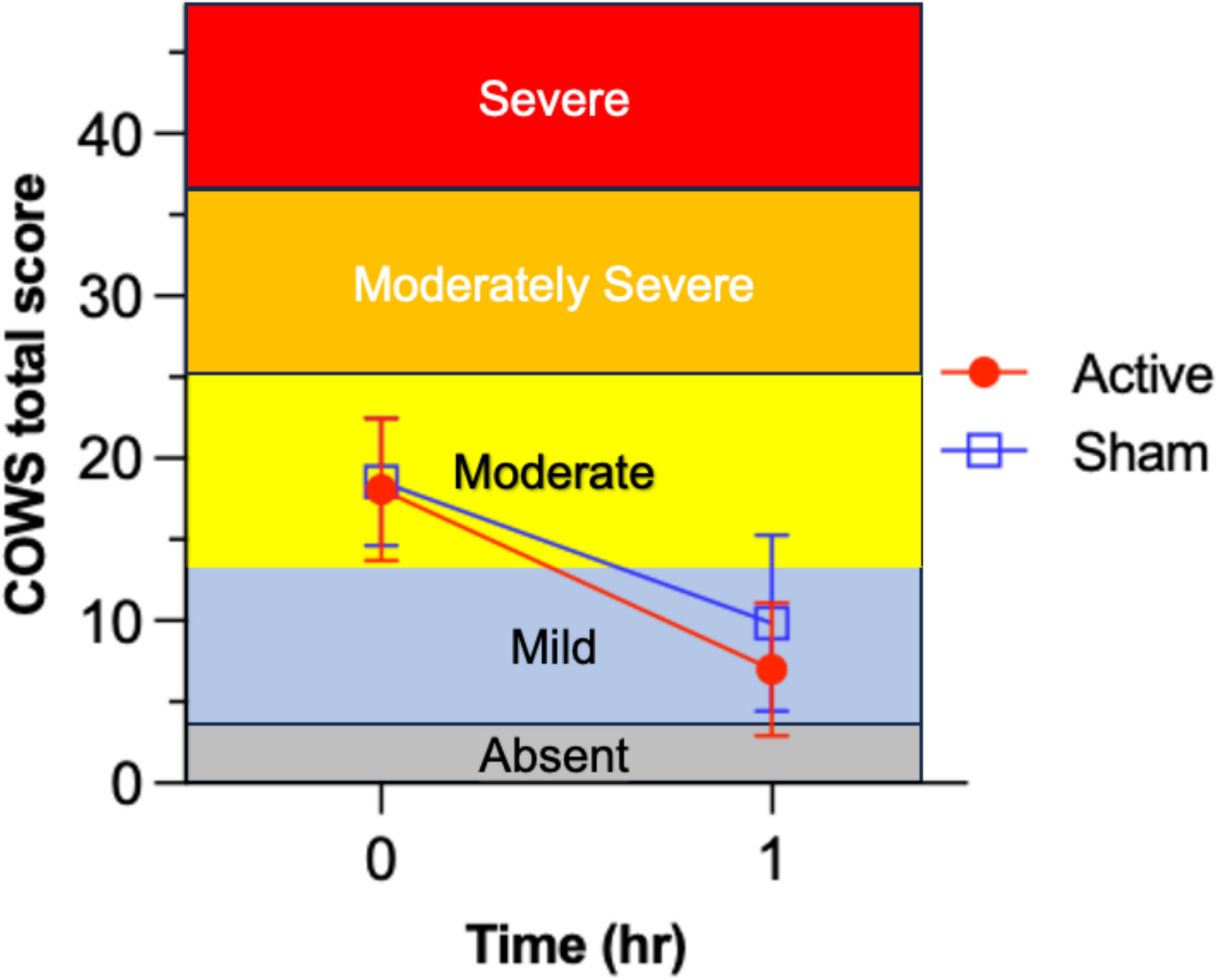
Test of efficacy endpoint for reduction of Clinical Opiate Withdrawal Scale (COWS) scores from before (t=0) to 1-hr after device utilization in the active device group (*n*= 52) and sham control group (*n*= 55). Comparison of mean ( ± 1 SD) opioid withdrawal symptom severity change from baseline (*t*= 0 hr) to 1-hr after starting active NET vs. sham device stimulation. Different horizontal color bands indicate the relative ordering in clinical severity for the COWS total score. Active NET stimulation led to significantly greater 1-hr decrease in opioid withdrawal severity than sham, from clinically moderate to mild levels (on average).

**Figure 2** presents results for the primary outcome analysis. Mean (± 1 SD) COWS score in the active NET group decreased from baseline (18.1 ± 4.4) to 1-hr (7.0 ± 4.1); this 61.3% decrease was highly significant, *t*= 15.46, *p*<.001, Cohen’s *d*= 2.14, exceeding the pre-specified 15% criterion. For the secondary outcome, mean ( ± 1 SD) change in COWS total score was -11.1 (5.2) for the active NET group and -8.8 (6.3) for the sham group. The independent *t*-test of the group difference in mean change in opioid withdrawal symptom severity score was significant, *t*= -2.14, *df*= 105, *p*= .035, Cohen’s *d*= -.41. These results are consistent with a superior outcome in the active *vs*. sham group, although the significance level of the test (*p*= .035) was not less than the Bonferroni-adjusted *α= .025.*

The analysis was repeated for the per-protocol group to assess sensitivity of the findings to completing the 1-hr intervention. The mean difference between the per-protocol active (n= 49) and sham (n= 54) groups in COWS baseline to 1-hr change was compared with two-tailed α= 0.025 to correct for multiple comparisons. The mean (SD) change in COWS score was -11.08 (4.83) and -8.94 (6.17) for the active and sham groups, respectively. Again, the group difference was significant, t= 4.26, df= 101, p= .042, however, the significance level of the test (p= .035) was not less than the Bonferroni-adjusted α= .025.

In a pre-specified analysis, aligned with the FDA 501(k) pathway for demonstrating “substantial equivalence” of the intervention to the predicate device, we calculated the group percentage difference in baseline to 1-hr COWS total scores. The mean (SD) percentage reduction in COWS score was -61.7% (4.9%) for the active NET group and -45.8% (8.9%) for the sham group. Independent t-test of the active vs. sham group comparison of percent reduction in withdrawal symptom severity was highly significant, t= 3.15, df= 100, p= .002, d= -.61 (moderate effect size).

In supporting analysis, we calculated the percentage of participants in each group that met the “clinically meaningful” criterion for >15% reduction in COWS total score from baseline to 1 hr. In the active NET group, 98.1% demonstrated a 15% or greater COWS score reduction and in the sham group, 83.6% had a 15% or greater COWS score reduction. Fisher’s Exact Test indicated that these proportions were significantly different, *p*= .016.

**Figure 3:**
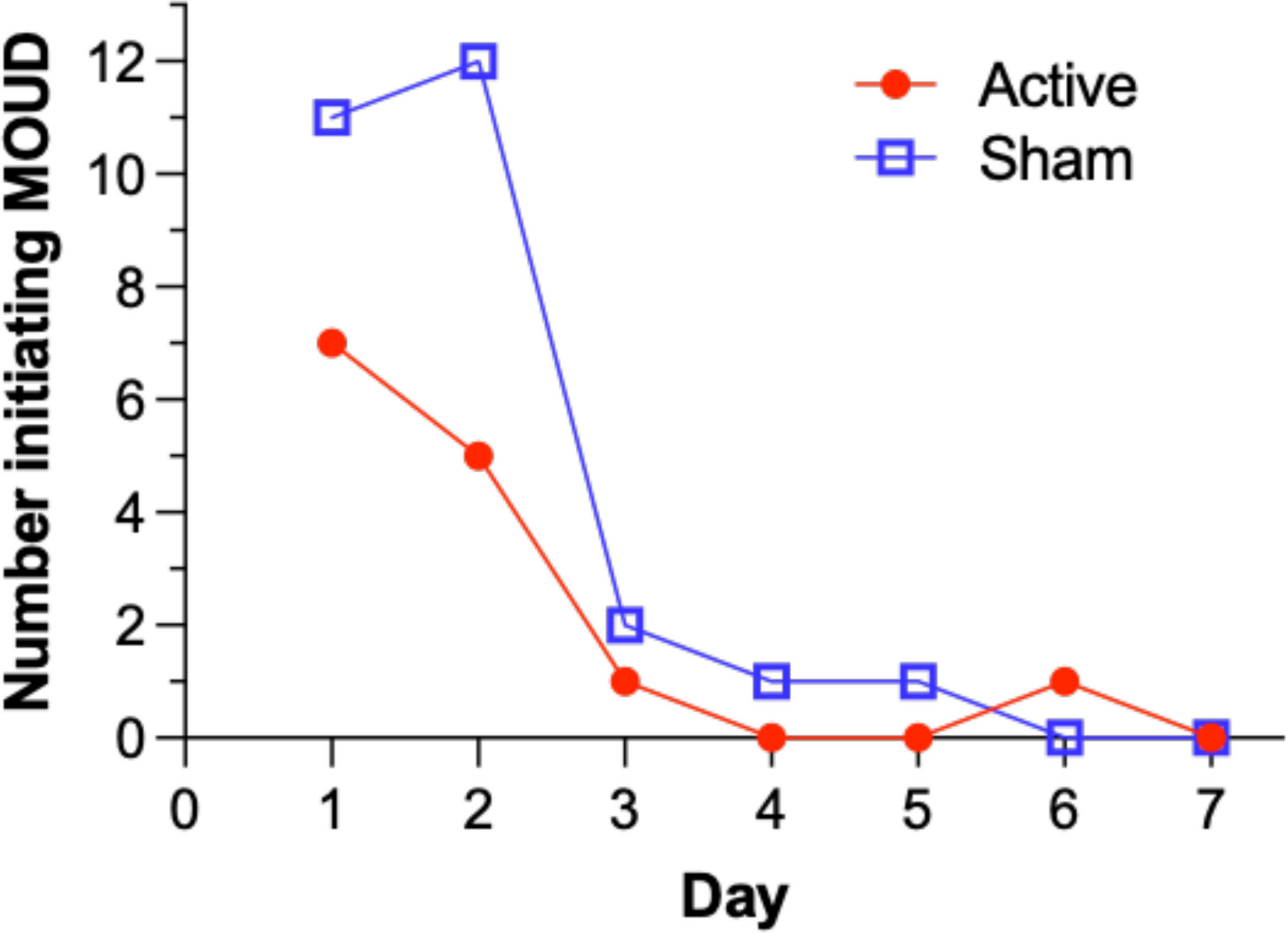
Number of participants requesting initiation of Medication for Opioid Use Disorder (MOUD) over inpatient days in the intent-to-treat population.

Participants could experience clinical deterioration during the opioid discontinuation process and could request MOUD at any time. Upon delivery of MOUD, use of the device was discontinued, but they were still included in the ITT analysis according to the group to which they were originally assigned. **Figure 3** illustrates the number of participants in each arm who requested MOUD during the inpatient period. A significantly lower proportion of participants in the active arm (26%, 14/53) requested inpatient MOUD than participants in the sham arm (49%, 27/55), χ2 (1, *n*=108)= 5.89, *p*= .015, indicating a significant difference between the active and sham groups. Most initiation of MOUD occurred in the first two days.

**Figure 4:**
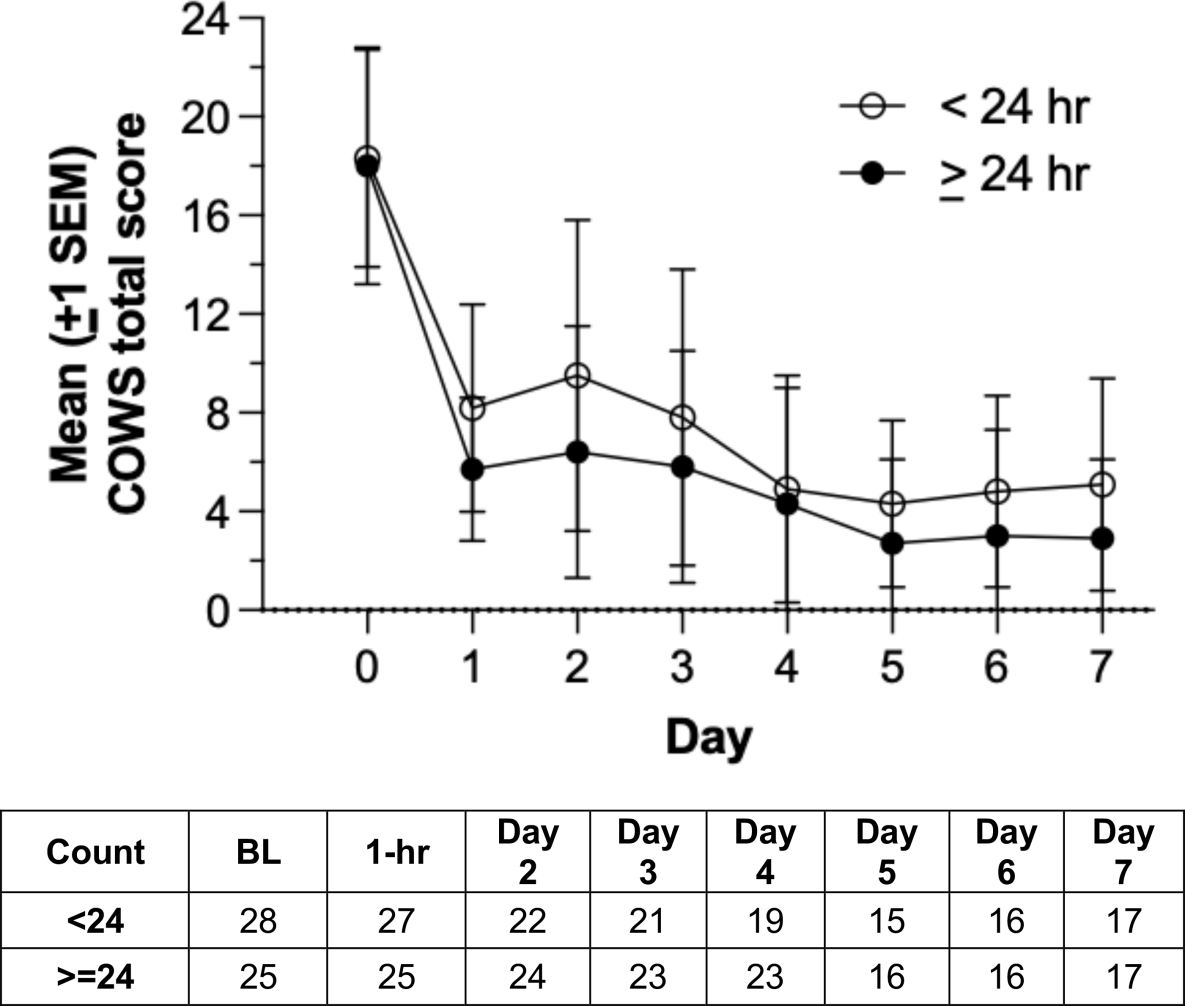
Mean Clinical Opiate Withdrawal Scale (COWS) scores over inpatient days in the active group, stratified by duration of device utilization (<24 hr vs. ≥24 hr). Group sizes are shown in the accompanying table.

**Figure 4** illustrates mean opioid withdrawal severity scores across inpatient treatment days in the active group, stratified by duration of device utilization (<24 hr *vs*. ≥24 hr). Mean (SD) COWS score reductions from baseline to day 7 were -15.2 (6.3) and -11.2 (5.6) for active participants receiving >24 hr and <24 hr of stimulation, respectively. Participants in the active arm receiving >24 hr of stimulation had significantly larger COWS score reductions than those receiving <24 hr of stimulation, *t*= 2.03, *df*= 37, *p*= .046. Active arm participants with device utilization >24 hr were significantly less likely to use MOUD on the inpatient unit (8.0%, 2/25) than those with <24 hr device utilization (42.9%, 12/28), χ2 (1, *n*= 53)= 8.26, *p*= .004.

**Supplementary Table S3A** compares opioid withdrawal suppression with the NET Device active arm in this study, relative to the predicate device (Sparrow) and another reference device (The Bridge) reported in prior studies. In brief, the NET Device demonstrated substantial equivalence in terms of efficacy with these prior devices.

**Supplementary Table S3B** compares the study population in this study relative to those other studies. The participants in this study had much higher rates of fentanyl exposure, and comparable rates of psychostimulant exposure, to studies using the predicate device.

### Safety

There were no reports of electrical, mechanical, or thermal hazards, injury, skin irritation, dizziness, or vertigo. One patient in the active device arm reported headaches. No patients in the active arm reported worsening opioid withdrawal discomfort.

### Supportive measures

#### Perception of device treatment

Mean (SD; 95% CI) device treatment perception ratings were 3.81 (0.99; 3.53–4.08) and 2.98 (1.23; 2.65–3.31) for active and sham groups, respectively (**Supplementary Figure S4**). Median ratings were 4 and 3, respectively. The groups significantly differed, Mann-Whitney *U*= 905.5, two-tailed *p*<.001. The James Blinding Index was 0.58 (CI 95%: 0.51–0.65). Thus, at 1-hr post-stimulation, the blind was moderately maintained.

#### Acceptability of device

##### Device satisfaction

After categorizing 1-5 Likert ratings into 1-3 (unsatisfied or ambivalent) and 4-5 (somewhat or very satisfied), 51.9% of the active group was highly satisfied compared to 43.6% of the sham group (χ^2^ = 0.74, *df*= 1, *p*= .391). Thus, device satisfaction did not significantly differ by active *vs*. sham treatment.

##### Willingness to use device

In the entire sample (*n*= 107, *n*= 1 missing), 71.7% (*n*= 77) answered 4 or 5 on the 1-5 Likert scale (somewhat or very willing to use the device) whereas 28% (*n*= 30) were less enthusiastic (unwilling or ambivalent). For the active NET group, responses were skewed such that those receiving NET stimulation were significantly more likely to answer 4 or 5 (80.8%) compared to the sham group (63.6%), χ^2^ = 3.89, *df*= 1, *p*= .049.

##### Willingness to recommend device to others

For the active NET group, responses were skewed. Thus, data were categorized into two groups, 1–3 (dissatisfied or ambivalent; 34.6% of respondents; *n*= 18) *vs*. 4–5 (somewhat or very satisfied; 65.4% of respondents; *n*= 34). Using Welch’s test, participants were significantly more likely to recommend the device at 1-hr post-stimulation if they had lower COWS withdrawal severity scores at that same time point, *t*= 2.39, *df*= 26.52, *p*= .024. For the sham group at 1-hr post-stimulation, 41.8% (23/55) were dissatisfied or ambivalent and 58.2% (32/55) were somewhat or very satisfied.

##### Duration of device utilization

**Figure 5:**
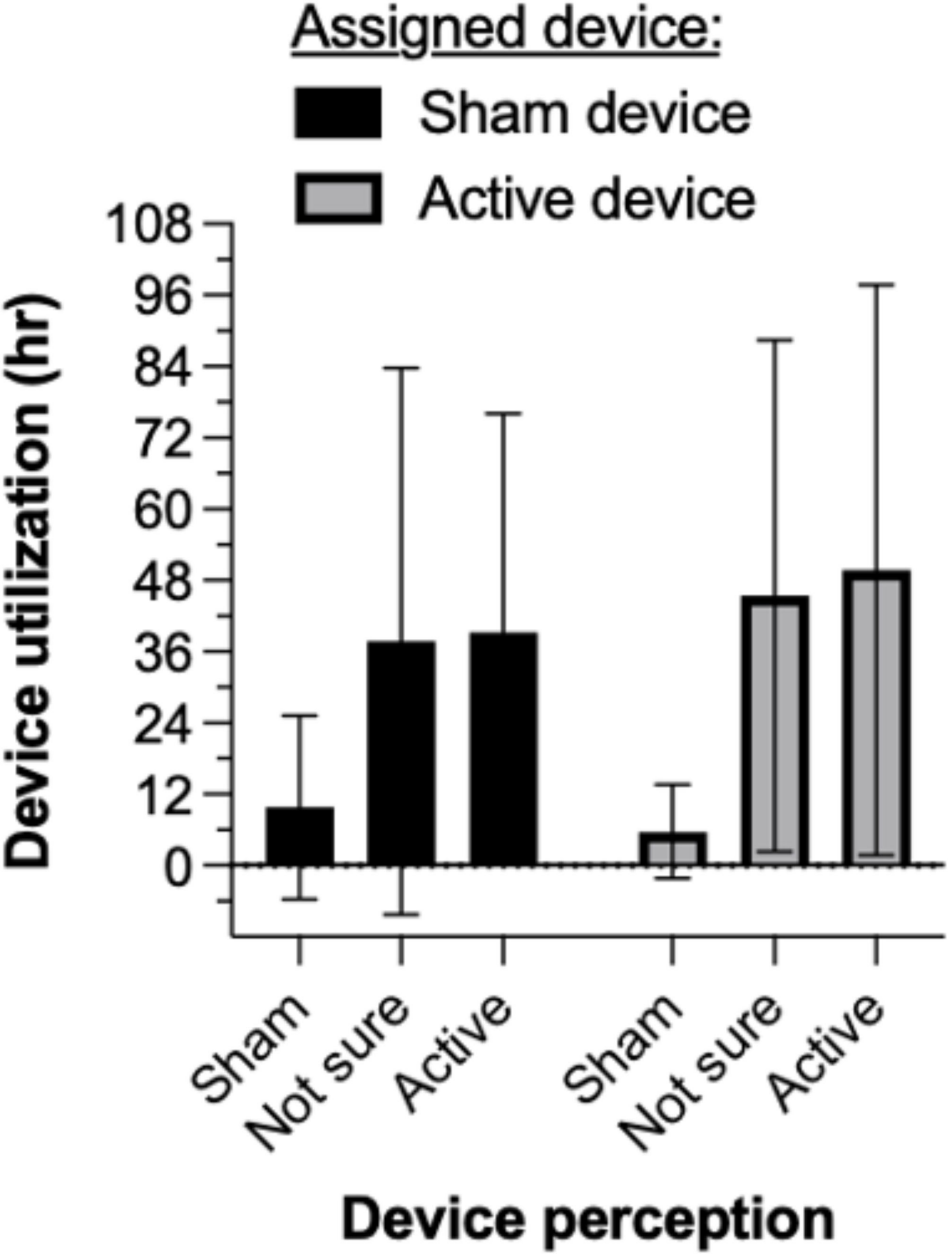
Mean ( ± 1 SD) duration of device utilization. Participants who perceived they were receiving active stimulation, regardless of their device assignment, used the device about 4-fold times longer (36-48 hr on average) than participants who perceived they were receiving sham stimulation (<10 hr on average).

Mean (SD) duration of device utilization was 43.9 (46.2) *vs*. 30.0 (39.2) hr in the active and sham groups, respectively. Due to higher variability in the sham group (due to 2 participants), non-parametric analysis was used. The group difference was statistically different, Kruskall-Wallis *H*= 7.14, *p*= .008. **Supplementary Figure S5** shows that participants who presented a fentanyl-positive urine sample at intake used their randomly assigned device (active or sham) numerically but not significantly longer than those who tested fentanyl-negative, both in the active NET treatment condition (52.9 [47.8] *vs*. 21.2 [29.8] hr) and in the sham condition (32.4 [40.1] *vs*. 24.2 [34.8] hr). **Figure 5** illustrates that participants who perceived they were receiving active stimulation, regardless of whether they received active or sham device assignment, used the device about 4-fold times longer (36-48 hr on average) than participants who perceived they were receiving sham stimulation (<10 hr on average). In the active NET group, device utilization across 5 inpatient days was associated with a sustained reduction in opioid withdrawal symptoms to average levels that were clinically mild (COWS score 5-12) or indicate no active withdrawal (COWS score <5).

#### Craving and mood scores, and clinical staff opinions

The **Supplementary Materials** report data on participants’ opioid craving scores (**Supplementary Figure S6)** changes in positive and negative affect scores (**Supplementary Figure S7**) and changes in depression, anxiety, and stress scores (**Supplementary Figure S8**) as well as clinical staff opinions of whether they would favor offering this treatment option (**Supplementary Figure S9**).

#### Summary of results

**Figure 6:**
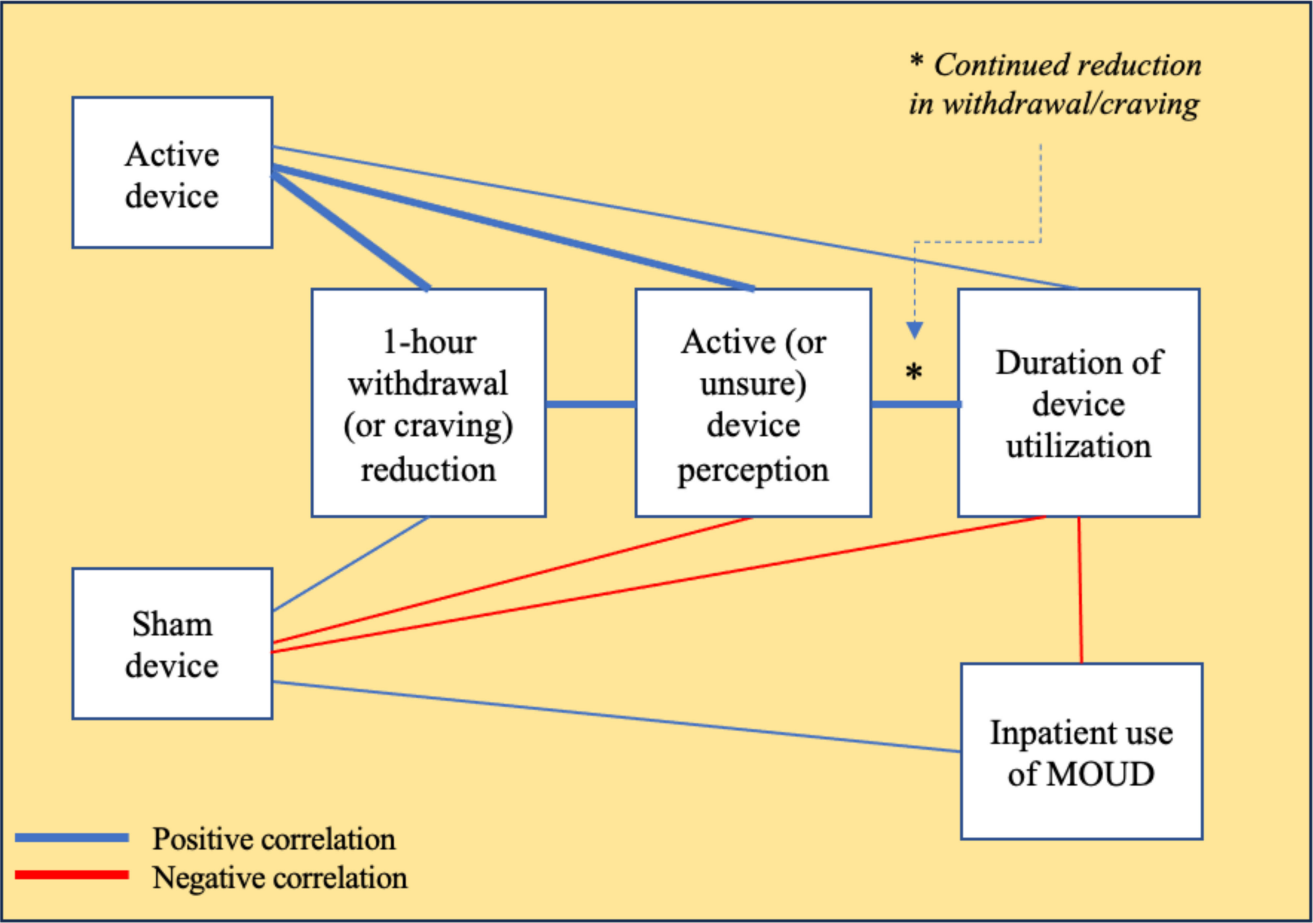
Summary of findings.

**Figure 6** summarizes significant interrelationships between various outcomes in relation to active *vs*. sham device assignment. Active device assignment was associated with greater reduction in opioid withdrawal symptom severity scores that, in turn, related to greater perception of receiving the active device, longer device utilization, and lower likelihood of using MOUD on the inpatient unit. In contrast, sham device assignment was associated with lesser-magnitude opioid withdrawal reduction that, in turn, was associated with lower perception of receiving the active device, shorter device utilization, and higher likelihood of using MOUD on the inpatient unit.

## Discussion

The illicit opioid use epidemic continues, current MOUD treatments are under-utilized, and some patients seek non-medication approaches to discontinue their illicit opioid use (22–27, 42, 43). Ethically responsible controlled trials are needed to evaluate the efficacy of medical devices for opioid discontinuation, while allowing use of OUD medications (e.g. if the patient changes his/her mind). We considered a study design where NET treatment is delivered (active and sham) as adjunctive therapy for participants receiving MOUD. Such a design would avoid exposing participants in the sham condition to ineffective treatment. Such a design, however, would expose the active population to stimulation concurrent with MOUD. This is contrary to the current recommended use of FDA-approved percutaneous nerve stimulators for substance use disorders (44). To minimize the impact of ineffective treatment on the sham population, we allowed participants to stop device treatment and request TAU at any time after one hour had elapsed.

Although the severity of withdrawal reduced from a moderate to a mild degree of severity in both the active and sham groups, the reduction was significantly greater by an average of 15.9% (61.7% in active group *vs* 45.8% in sham group). This large reduction in the sham group is not just a placebo effect. In an ITT analysis the groups are analyzed according to the group to which they were originally assigned, even if they are using medication to assist with opioid withdrawal symptoms (MOUD). Following the initial 1-hr efficacy evaluation window (during which MOUD was not allowed), 49% of the sham group converted to MOUD, compared to only about half that number (26%) in the NET treatment group. The reduction in opioid craving is very similar in both treatment groups for the same reason.

The NET Device has advantages over other tACS devices, including that it is self-administered by the patient, provides little or no sensation when active, and adjusts itself for variations in skin impedance and electrode conductance which could contribute to its high patient tolerability and efficacy. As evidence of device acceptability, we found that duration of device utilization exceeding 24 hr led to greater reduction in opioid withdrawal symptom severity over a 7-day period and to lower likelihood of using MOUD on the inpatient unit. Notably, participants in this study of the NET Device had much higher rates of fentanyl use than studies of prior devices, yet comparable efficacy was observed for withdrawal symptom reduction.

Random assignment to the active device led to greater reduction in opioid withdrawal symptom severity, as well as greater perception of receiving the active device, longer duration of device utilization, and reduced patient demand for MOUD. In contrast, random assignment to sham led to less reduction opioid withdrawal symptom severity that, in turn, correlated with lower perception of receiving the active device, shorter device utilization, and higher likelihood of using MOUD on the inpatient unit. Device perception ratings indicated that not all participants knew their device assignment, however, more participants assigned to active NET perceived it to be active stimulation, whereas participants assigned to sham were unsure.

Earlier studies demonstrated that transcranial electrostimulation of the type delivered by the NET Device can attenuate the severity of opioid withdrawal (45–49). Electrical neurostimulation is thought to produce frequency-dependent modulation of endogenous opioid, dopaminergic and serotonergic systems and the autonomic nervous system (50–52), which are dysregulated in the opioid-dependent state (53–59). It seems likely that self-titrated NET Device stimulation of these multiple interacting neurochemical systems may promote neuroplastic changes (60–63) that normalize functioning of these systems and support longer-lasting changes in drug-abstinence behavior. In future studies, we intend to evaluate the neurochemical mechanisms of action that may underlie the efficacy of NET. Understanding the mode of action of this intervention could provide useful data for determining whether it may complement other types of interventions.

The study has several limitations. First, the study population consisted of people willing to be admitted to a single, rural Kentucky-located residential treatment unit to discontinue their illicit opioid use; although this treatment facility draws from a wider geographic area, results may not generalize to every person seeking opioid discontinuation. Second, participants sought opioid discontinuation, at least initially, without the assistance of MOUD. This group tends to be highly motivated and, on average, experienced mild to moderate (rather than severe) opioid withdrawal symptoms. The residential setting, high motivation of individuals, and the option of MOUD are likely to have contributed to the high retention rate of 75% after 5 days. Third, there were relatively small differences in withdrawal symptom scores between active and sham arms. This study design did not establish a withdrawal symptom time course related to illicit opioid (typically fentanyl) discontinuation in the absence of active or sham device, thus, it is not possible to estimate the size of the placebo effect.

In conclusion, this pivotal randomized, sham-controlled trial of the NET Device found that active neurostimulation was associated with clinically meaningful (61%) reduction of opioid withdrawal symptom severity in virtually all (98%) participants, and significantly more so than sham stimulation. The FDA cleared the NET Device for marketing on May 29, 2024, with the following indication for use: “The NET Device is a transcutaneous alternating current stimulator (tACS) that is intended to be used in patients experiencing opioid withdrawal in conjunction with standard symptomatic medications and other therapies for opioid withdrawal symptoms under the supervision of trained clinical personnel.” In summary this device is a promising non-medication approach to treating opioid withdrawal, has the potential for use in a community setting, and will help towards managing the opioid epidemic.

## Data Availability

The datasets presented in this article are not readily available. Requests to access the data will be evaluated by the sponsor (NET Recovery Corp.) and provided if deemed appropriate. All study-related information will be regarded as confidential. Requests to access the datasets should be directed to JW, joe.winston@netrecovery.net.
