# Supplementary material for: A randomized, sham-controlled clinical trial to evaluate the NET Device™ for reducing withdrawal symptom severity during opioid discontinuation
Source: Front Psychiatry. 2025 Feb 19;16:1510428. doi: 10.3389/fpsyt.2025.1510428 (PMC11880230; doi:10.3389/fpsyt.2025.1510428)

Supplemental Materials

Supplemental Table 1.	Participant selection criteria.
Supplemental Table 2.	Description of ITT sample, stratified by treatment facility.
Supplemental Table 3A.	Comparisons of COWS withdrawal symptom score reductions from baseline between the NET Device (active treatment arm), the predicate device (Sparrow Therapy System; Spark Biomedical, Inc.), and a reference device (NSS-2 Bridge; Innovative Health Solutions, Inc.)
Supplemental Table 3B.	Comparisons of NET Device, predicate and reference device study populations for fentanyl-, methamphetamine-, and cocaine-positive urine data screens at baseline.
Supplemental Figure 1.	Study schema.
Supplemental Figure 2.	Schedule of activities.
Supplemental Figure 3.	NET Device.
Supplemental Figure 4.	Device treatment perception.
Supplemental Figure 5.	Association of pre-enrollment fentanyl use with outcomes.
Supplemental Figure 6.	Opioid Craving Scale (OCS) results.
Supplemental Figure 7.	Positive and negative affect change.
Supplemental Figure 8.	Change in depression, anxiety, and stress.
Supplemental Figure 9.	Clinical staff opinions.

Supplemental Table 1. Participant selection criteria

Inclusion Criteria	*Exclusion Criteria*
(1) Meet DSM-5 criteria for opioid use disorder (any severity level) alone or comorbid with stable medical diseases (except for certain medications below)<br>(2) Stated desire to be opioid abstinent without medication for opioid use disorder<br>(3) Initiating opioid discontinuation at study site<br>(4) In good general health as evidenced by medical history<br>(5) Male or female, aged 18-65 years<br>(6) Provision of signed and dated informed consent and HIPAA authorization form<br>(7) Stated willingness to comply with all study procedures and availability for the duration of the study (including inpatient stay and participation in video assessments and remote drug screens for the duration of the 12-week outpatient study period)<br>(8) Use of highly effective contraception for at least 1 month prior to screening and agreement to use such a method for the duration of the study<br>(9) Treatment will not start until the COWS total score is 13 or greater (at least moderate withdrawal); thus, a consented participant could be dis-enrolled	(1) Acute/unstable illness: conditions making it unsafe to participate<br>(2) Chronic illnesses: primarily seizures and dementing illnesses, including medications for these neurological conditions<br>(3) Current serious psychiatric disease: psychosis, bipolar disorder<br>(4) Current requirement to detoxify for alcohol or benzodiazepines<br>(5) Current use of anxiolytics, hypnotics (prescription and over-the-counter), antidepressants, anticonvulsants, sedating H1 antihistamines (non-sedating second generation H4 antihistamines are allowed), prescription and over-the-counter stimulants<br>(6) Current diagnosis other than opioid use disorder requiring chronic opioid treatment<br>(7) Presence of a cardiac pacemaker<br>(8) Pregnancy or lactation. Females who do not agree to sexual abstinence or are heterosexually active and not using (self-report) medically approved birth control measures (sterilization, tubal ligation, oral/depot contraceptives, abstinence, intrauterine device, barrier method such as condom/foam, or a cervical cap combined with a spermicide), are not eligible<br>(9) Receiving extended-release buprenorphine within 300 days of enrollment

Supplemental Table 2. Description of the ITT sample (*N*=108), by de-identified treatment facility.

	**Total sample**	**Facility 1 (male)**	**Facility 2 (male)**	**Facility 3 (female)**	**Facility 4 (female)**
Sex identity					
Female	44 (40.7%)	0	0	35 (100%)	9 (100%)
Male	64 (59.3%)	38 (100%)	26 (100%)	0	0
Missing	0				
Race					
White	97 (89.8%)	35 (92.1%)	23 (88.5%)	31 (88.6%)	8 (88.9)
Black	6 (5.6%)	1 (2.6%)	3 (11.5%)	3 (8.6%)	0
Native American	2	1		1	
Pacific Islander	1	1			
Multiple	2			1	1
White, Arabic	1			1	
White, Black	1				1
Other (Native American, Pacific Islander, Multiple)	5 (4.6%)	2 (5.3%)	0	2 (5.7%)	1 (11.1%)
Missing	0				
Ethnicity					
Hispanic	3 (2.8%)	1 (2.6%)	0	1 (2.9%)	1 (11.1%)
Not Hispanic	103 (95.4%)	36 (94.7%)	26 (100%)	33 (94.3%)	8 (88.9%)
Unknown	2 (1.9%)	1 (2.6%)	0	1 (2.9%)	0
Age					
Mean, SD	34.05, 8.4	34.47, 7.46	34.62, 8.95	33.80 8.76	31.56, 9.75
Median, IQR	34.0, 12	35.0, 12	34.0, 10	32.0, 12	33.0, 18
Range	18-62	19-53	18-62	18-57	19-46
Missing	0	0	0	0	0
Pediatric age					
Age 18-20	5 (4.6%)	1 (2.6%)	1 (3.8%)	2 (5.7%)	1 (11.1%)
Age 21+	103 (95.4%)	37 (97.4%)	25 (96.2%)	33 (94.3%)	8 (88.9%)
Missing	0	0	0	0	0
Primary substance use disorder					
OUD	88 (81.5%)	35 (92.1%)	22 (84.6%)	24 (68.6%)	7 (77.8%)
other	20 (18.5%)	3 (7.9%)	4 (15.4%)	11 (31.4%)	2 (22.2%)
Polysubstance use disorder					
Yes	101 (93.5%)	34 (89.5%)	26 (100%)	33 (94.3%)	8 (88.8%)
No	7 (6.5%)	4 (10.5%)	0	2 (5.7%)	1 (11.1%)
Length of residential stay					
<20 days	51 (47.7%)	20 (52.6%)	12 (46.2%)	12 (35.3%)	7 (77.8%)
20 days or more	56 (52.3%)	18 (47.4%)	14 (53.8%)	22 (64.7%)	2 (22.2%)
Missing	1			1	

Supplemental Table 3A

This table presents 1-hour and 5-day comparisons of COWS score reduction from baseline between the NET Device (active treatment arm), the predicate device (Sparrow), and the reference device (NSS-2 Bridge, DEN 170018). The NET Device percentage reduction from baseline is greater than the predicate device and lower than the reference device. Note that the reference device study was retrospective and without a control group.

	Baseline		1-hour			5-days		
	Participants	COWS Mean (SD)	Participants	COWS Mean (SD)	% Reduction to Baseline	Participants	COWS Mean (SD)	% Reduction to Baseline
**NET Device**	n=53	18.1 (4.4)	n=52	7.0 (4.1)	61.3%	n=40	3.5 (3.4)	81.0%
**Sparrow**[1]	n=26	15.6 (2.7)	n=26	7.9 ( )	49.4%	n=14		
**Sparrow**[2]	n=31	15.3 ( )	n=31	8.3 (4.7)	45.9%	n=13	6.9 (5.0)	55.1%
**Bridge**[3,4]	n=73	20.1 (6.1)	n=71	3.1 (3.4)	84.6%	n=33	0.6 ( )	97.0%

[1] K201873 510(k) Summary for Sparrow Therapy System.

[2] Tirado CF, Washburn SN, Covalin A, et al (2022) Delivering transcutaneous auricular neurostimulation (tAN) to improve symptoms associated with opioid withdrawal: results from a prospective clinical trial. *Bioelectronic Med* 8: 12. DOI: 10.1186/s42234-022-00095-x

[3] DEN170018 De Novo Classification Request for NSS-2 Bridge.

[4] Miranda A, Taca A (2018) Neuromodulation with percutaneous electrical nerve field stimulation is associated with reduction in signs and symptoms of opioid withdrawal: a multisite, retrospective assessment. *Am J Drug Alcohol Abuse* 44(1): 56-63. DOI: 10.1080/00952990.2017.1295459

Supplemental Table 3B

This table indicates that the NET Device study population had significantly higher prevalence of fentanyl, methamphetamine, and cocaine on admission as compared to the reference device studies, which may contribute to the difference in COWS reduction.

	Participants	Urine-positive for fentanyl	Urine-positive for methamphetamine	Urine-positive for cocaine
**NET Device**	N=53	72%	47%	11%
**Sparrow**	N=26	3%	40%	20%
**Bridge**	N=73	0%	0%	3%

Supplemental Figure 1. Study schema

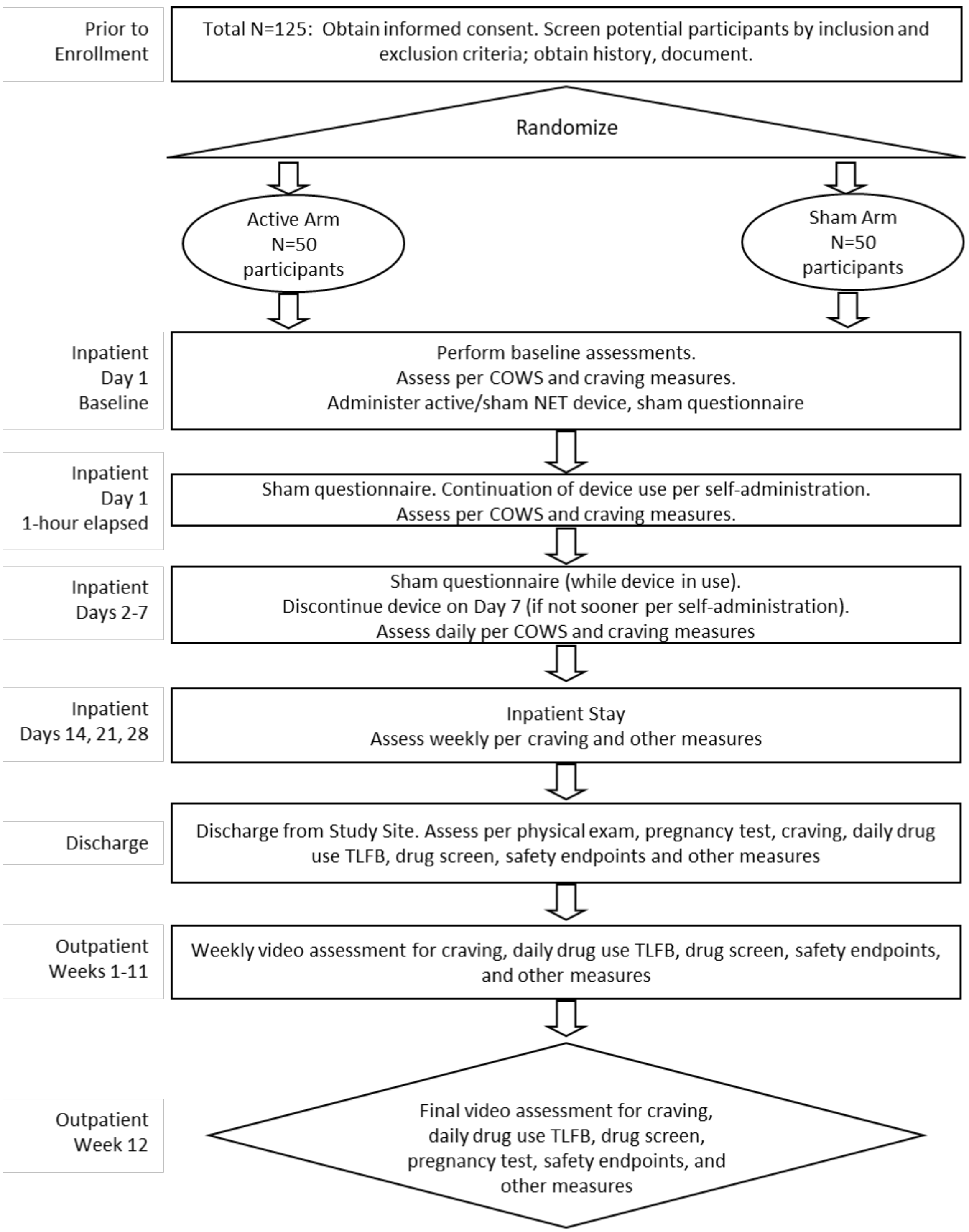

Notes: "NET", NeuroElectric Therapy. "COWS", Clinical Opiate Withdrawal Scale. "TLFB", Timeline Followback

Supplemental Figure 2. Schedule of Activities

Schedule of Activities (SoA)	Pre-Screening	Screening	Inpatient					Outpatient	
Study Week(s)	Week -1	Week 1				Weeks 2-3	Week 4	Weeks 5-15	Week 16
Inpatient Study Day(s)		Day 1	Day 1, Baseline	Day 1, One Hour Elapsed	Days 2-7	Day 14 Day 21	Day 28 (Discharge from Site)		
Outpatient Test Weeks								OP Wk 1-11 each Day 7	OP Wk 12 Day 7
Study Site Admission		X							
Study Site Discharge							X		
End of Study									X
Informed Consent	X	X[3]							
Demographics		X							
Medical and Psychiatric History		MIA[3]							
Drug Use History		MIA[3]							
Physical Exam (including height and weight)		MIA[3]							
Vital Signs (BP, HR, RR, O2)		MIA[3]							
Drug Screen		MIA[1,3]					X	X	X
Contraception, Pregnancy Test, Menstrual Cycle		X					X	X	X
Drug Use Timeline Followback Interview (TLFB)								X	X
Eligibility (Inclusion/Exclusion)		X							
Contact Data		X					X		
Randomization and Stratification		X							
COWS			X[2]	X	X				
Administer Active/Sham Treatment (self-administration [sa])			X	sa	sa				
Opioid Craving Scale (OCS)			X	X	X	X	X	X	X
Treatment Perception (while device in use [sa])				X	sa				
Device Satisfaction Scale (while device in use [sa])				X	sa				
Device Tolerability (while device in use [sa])				X	sa				
Dass21: Depression, Anxiety and Stress			X				X		X
PANAS-SF: Positive and Negative Affect Scale (Short Form)			X	X	X				
Epworth Sleepiness Scale			X				X		X
Concomitant Medications				X	X	X	X	X	X
Adverse Events			X	X	X	X	X	X	X
Device Deficiencies				X	sa				
SF-12 Health Related Quality of Life (Short Form)							X		X

[1] This drug screen is wider ranging to collect stratification information

[2] Must be >13 for inclusion on Day 1

[3] Paper (non-electronic) source data

"MIA" is Study Site Medical Intake Assessment

Notes: "OP", outpatient. "Wk", week. "sa", self-administered. "BP", blood pressure. "COWS", Clinical Opiate Withdrawal Scale, "HR", heart rate. "RR", respiration rate. "O2", oxygen saturation. "MIA", study site medical intake assessment.

Supplemental Figure 3. NET Device

The NET Device is a non-invasive, battery-powered, portable, re-usable, prescription device designed to provide bilateral, transcranial, transcutaneous, alternating current stimulation (tACS) to reduce symptoms of opioid withdrawal under the supervision of trained clinical personnel in a controlled setting (e.g. residential treatment, hospital).

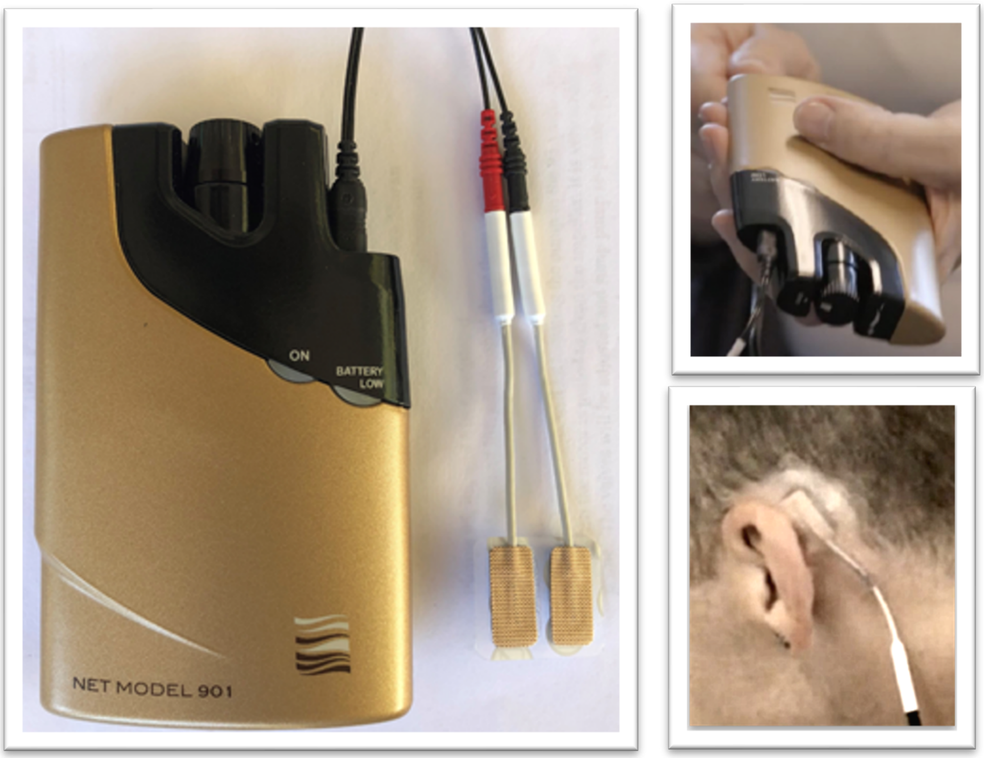

The system is comprised of one component (the NET Device), accessories (patient leads, electrodes, USB cable), and software (the clinician application).

Supplemental Figure 4. Mean (±1 SD) device treatment perception (X = mean, horizontal bar = median), showing that the study blind was moderately maintained.

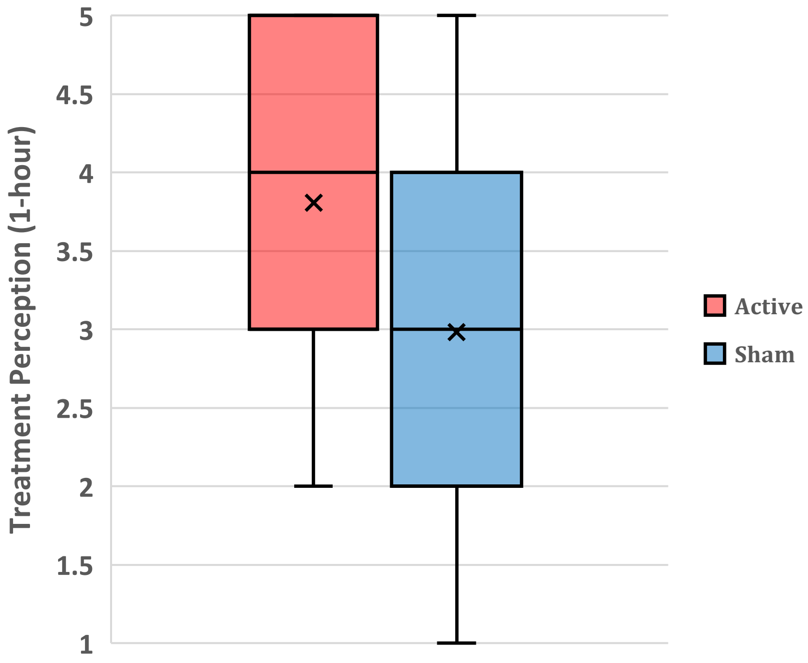

Supplemental Figure 5. Association of pre-enrollment fentanyl use with primary and secondary outcomes.

At screening this sample had very high rates of recent fentanyl exposure (77 of 108; 71.3%) based on urine drug screening (UDS). This positive drug-test rate is consistent with current rates of fentanyl-related overdose toxicological findings in Kentucky (Kentucky Office of Drug Control Policy, 2022). Fentanyl exposure was evenly distributed across active and sham groups.

Participants with fentanyl-positive UDS at screening had mean COWS withdrawal scores that were numerically, but not significantly, higher at baseline and at 1-hr than participants with fentanyl-negative UDS; there was no difference in COWS change scores from baseline to 1-hr for fentanyl-positive vs. fentanyl-negative UDS groups.

As shown in the figure, fentanyl UDS-positive participants used the device for a numerically (mean ± SD) but not significantly longer period than fentanyl UDS-negative participants, both in the active NET treatment condition (52.9 ± 47.8 vs. 21.2 ± 29.8 hr) and in the sham condition (32.4 ± 40.1 vs. 24.2 ± 34.8 hr).

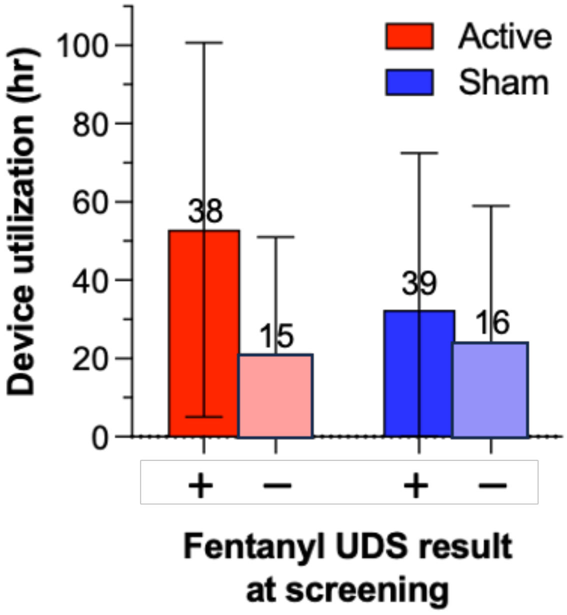

Supplemental Figure 6. Opioid Craving Scale (OCS)

The figure shows mean (± 1 SE) OCS total craving scores during inpatient days. Relative to baseline (t=0), craving scores decreasing monotonically across days of the inpatient stay. The two groups did not significantly differ in craving. There was substantial missing data after the first few inpatient days.

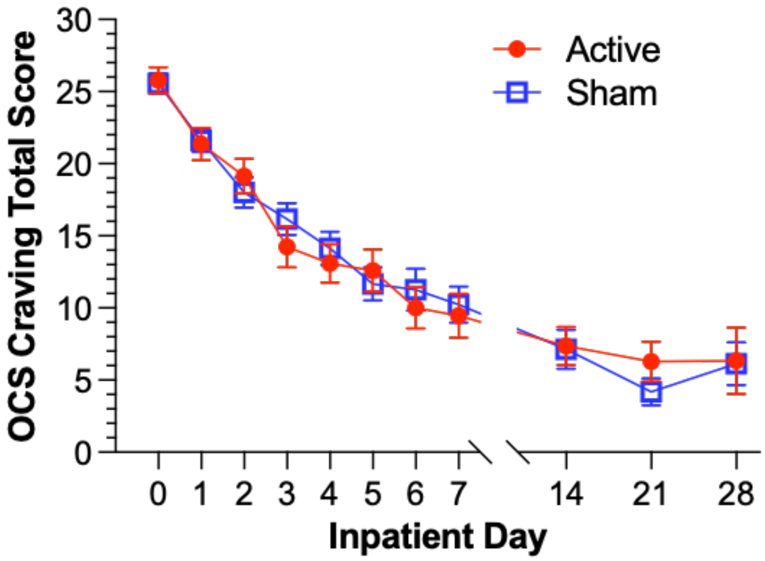

Supplemental Figure 7. Positive and negative affect change

Positive and Negative Affect Scale (PANAS short form) measures the extent of experiencing positive and negative symptoms modified to cover time "right now". This figure shows significant differences (t= 1.98, 2-sided p= 0.05) for change in positive affect after 1-hr (mean, SD) between active (3.60, 4.17) and sham (1.95, 4.66) groups. Negative affect change does not significantly differ between active and sham groups.

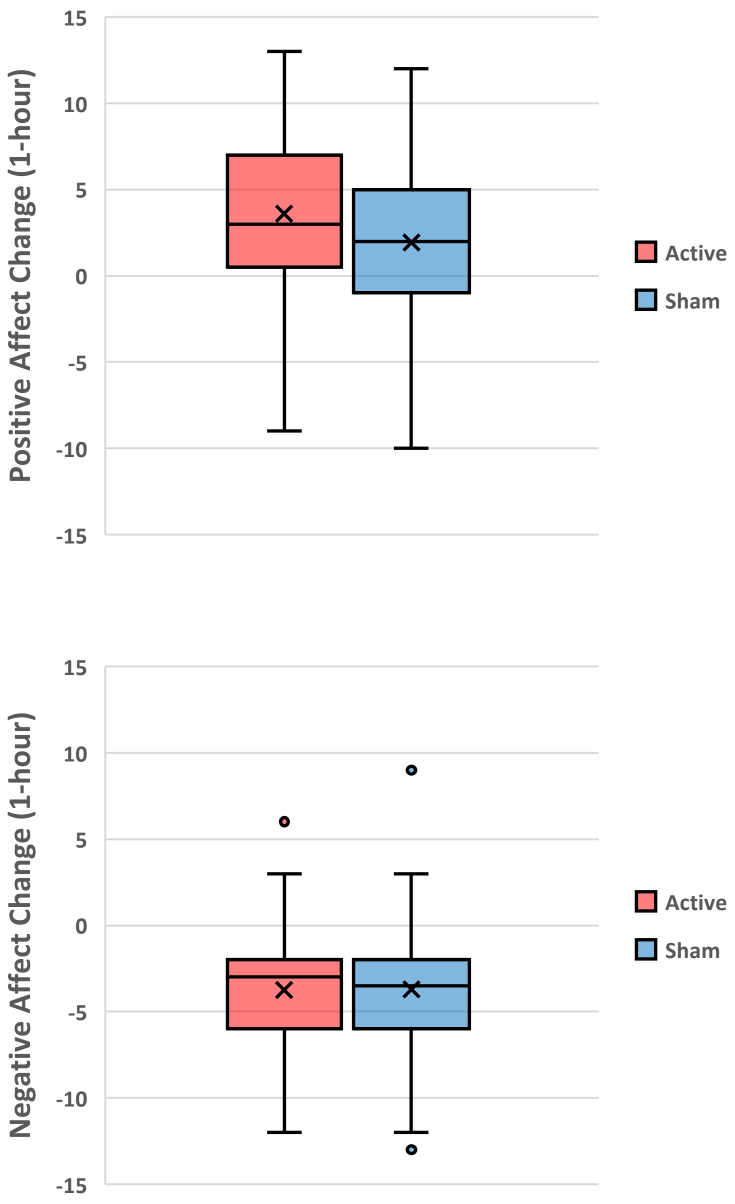

Supplemental Figure 8. Change in depression, anxiety, and stress

Depression Anxiety Stress Scale-21 (DASS-21) is a short version of a 42-item self-report instrument designed to measure three related negative emotional states: depression, anxiety, and tension/stress. This figure shows significant ($p < .001$) reductions in all three subscales (negative change scores) from baseline to inpatient discharge, but there were no significant differences between active and sham groups (adjacent bars) for any of these three subscales.

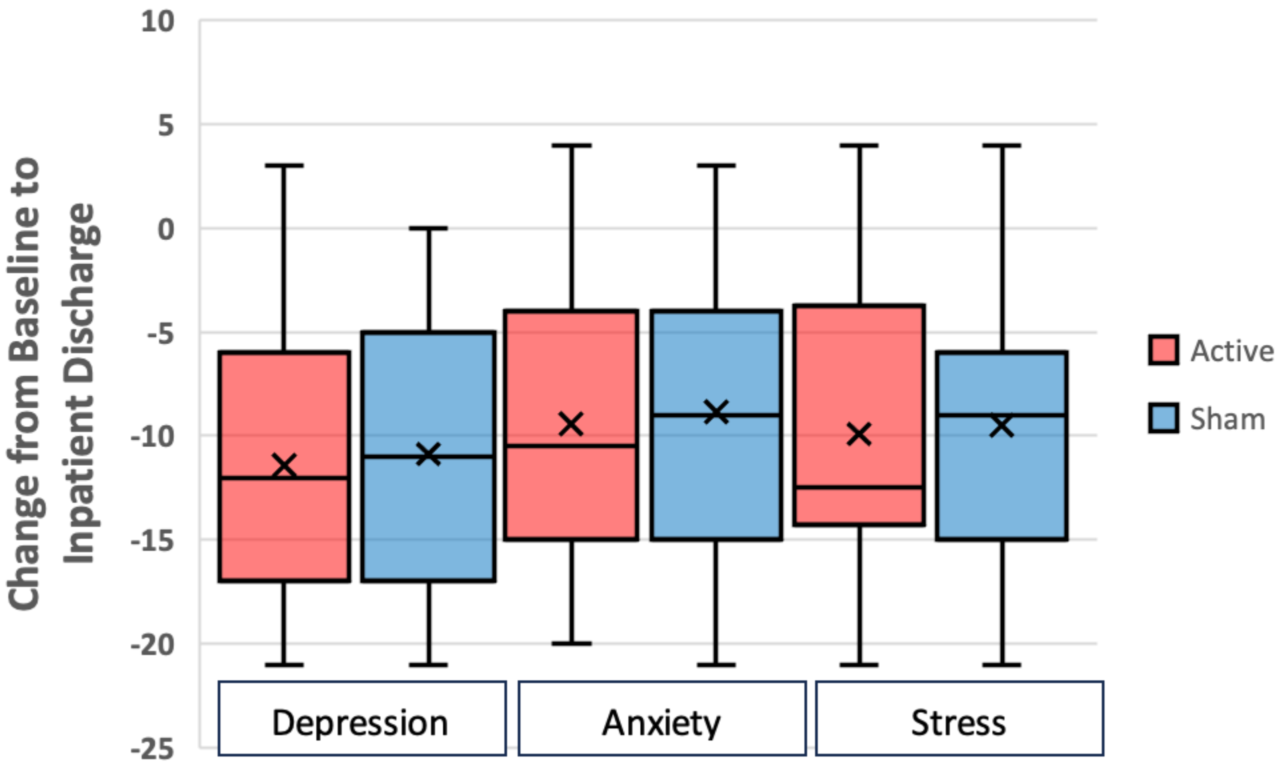

Supplemental Figure 9. Clinical staff opinions

Anonymous assessments of the NET Device (without knowledge of active vs. sham assignment) were provided by 15 treatment staff members who had observed this approach. Thirteen of the 15 respondents (87%) reported they were ‘very likely’ or ‘extremely likely’ to favor offering the NET treatment as part of their standard clinical protocol.

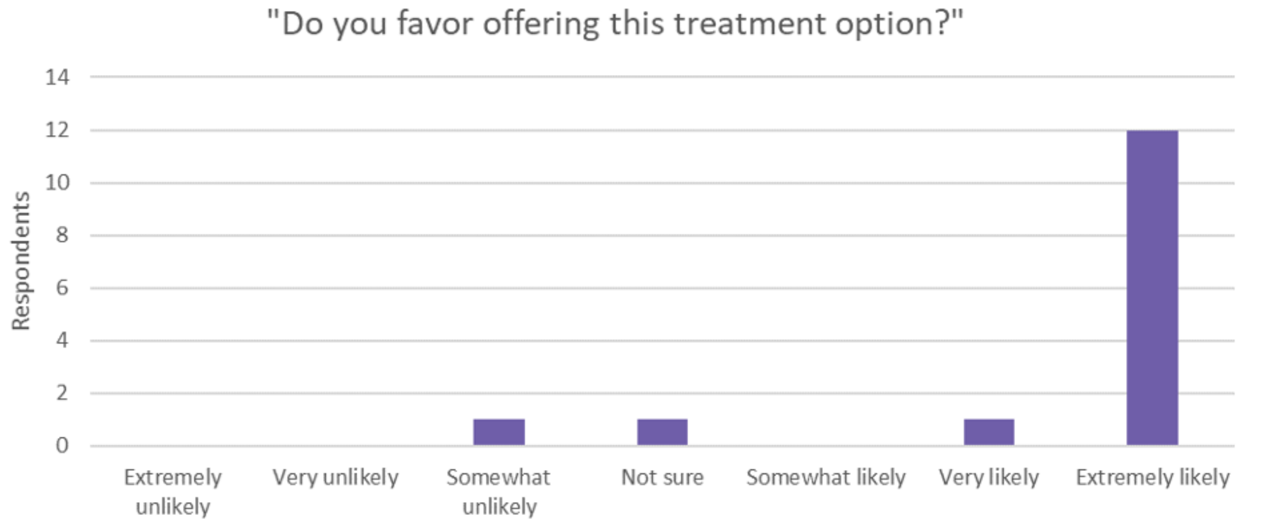

Supplement: Supplementary file 1 [file DataSheet1.pdf]
